# HIV understanding, experiences and perceptions of HIV-positive men who have sex with men in Amazonian Peru: a qualitative study

**DOI:** 10.1186/s12889-020-08745-y

**Published:** 2020-05-19

**Authors:** Jasmine Tattsbridge, Connie Wiskin, Gilles de Wildt, Anna Clavé Llavall, César Ramal-Asayag

**Affiliations:** 1 College of Medical and Dental Sciences, University of Birmingham, Birmingham, B15 2TT UK; 2 Department of Infectious Diseases, Regional Hospital of Loreto, Iquitos, Peru; 3 Department of Clinical Sciences, Universidad Nacional de la Amazonia Peruana, Iquitos, Peru

**Keywords:** HIV, Antiretroviral therapy, Men who have sex with men, Qualitative, Peru

## Abstract

**Background:**

HIV-related incidence and mortality is increasing across Peru, with highest mortality rates recorded in the Amazonian region of Loreto. This epidemic is concentrated in men who have sex with men, a population with 14% HIV treatment adherence despite free national provision. This study investigates barriers and facilitators to following healthcare advice through experiences and perceptions of HIV-positive men who have sex with men and healthcare professionals in Loreto.

**Methods:**

Twenty qualitative interviews with HIV-positive men who have sex with men and one focus group with HIV-specialist healthcare professionals were conducted in Loreto, January–February 2019. Interviews were transcribed per verbatim. Thematic content analysis and deviant case analysis were used.

**Results:**

A culture of isolation and discrimination was identified, propagated by poor public knowledge surrounding HIV transmission and treatment. Employment potential was hampered and 7/20 patients had suicidal thoughts post-diagnosis. Barriers to care included: shame, depression, travel cost/times, a preference for traditional plant-based medicine and side-effects of antiretroviral therapy. Facilitators included: education, family and clinic support, disease acceptance and lifestyle changes.

**Conclusion:**

More effective, focussed community education and workplace discrimination investigations are recommended to reduce stigma and increase adherence to treatment in this population.

## Background

HIV remains a significant local and global health problem, with an estimated 36.9 million infected worldwide [1]. The majority of cases are in low and middle-income countries, resulting in national governmental and non-governmental control efforts as well as international support from agencies including UNAIDs and the World Bank [1, 2]. Across South America there are still significant barriers in achieving optimal care for people living with HIV including stigma, discrimination, misinformed health beliefs, economic limitations and administrative barriers [3, 4]. Stigma has been shown to disproportionately affect men who have sex with men, a group that also have reduced access to care [5–9].

Antiretroviral therapy (ART) has been shown to prevent HIV transmission through condomless sex when HIV viral load is undetectable, achieved by strict adherence [10]. By 2020, UNAIDS aims to diagnose 90% of HIV-positive people, provide ART to 90% of these and achieve viral load suppression in 90% of those treated worldwide [11]. Adherence to ART in South American countries like Peru is likely to be below levels needed for viral suppression which may contribute to increasing HIV incidence and mortality rates [12, 13].

Peru is a lower-middle income country with approximately 70,000 people living with HIV [14, 15]. Prevalence has risen by 24% since 2010, and AIDS-related deaths have increased by 14% [14]. HIV is predominantly spread through unprotected anal intercourse [16]. The epidemic concentrates in key groups, with over 60% of new infections accounted for by transgender women and men who have sex with men [14, 17]. In 2017 the prevalence of HIV-positive men who have sex with men was 41 times greater (12.2%) than the general population (0.3%) [18]. An estimated 24% of HIV-positive men who have sex with men are aware of their diagnosis [14]. Of those that know their diagnosis: 15.6% have regular engagement with medical care; 13.6% are on ART; 12% have a supressed viral load [17].

The Ministry of Health in Peru has guidelines in place for the prevention, treatment and control of HIV, aligned with international standards [19]. Access to anti-retroviral therapy is free at the point of delivery. Based on researcher observations, patients collect their medication every 1–3 months from the HIV clinic where they are monitored by a multidisciplinary team. They receive education and psychological support in clinic, and are invited to attend small education meetings that are run infrequently at the hospital.

To reduce the Peruvian HIV epidemic UNAIDS have recommended the elimination of discrimination, known to have detrimental effects on treatment adherence [14, 20]. A 2018 cross-sectional report found that 47% of 600 people living with HIV across Peru had experienced some form of discrimination in the last year (Anamaria P, Silva-Santisteban A, Bustamante MJ: Índice de estigma y discriminación hacia las personas con en Perú, unpublished). A population survey of over 3000 Peruvians found that only 45% would consider hiring someone with HIV, despite work-based discrimination being illegal [21]. High-quality epidemiological data on discrimination is scant.

The Amazonian region of Loreto is a key area for HIV transmission, with the second highest prevalence of HIV in men who have sex with men (11–14%), after Lima (12–22%) [22]. Loreto has the highest regional adjusted HIV mortality rates (21.2 per 100,000) and years of potential life lost from HIV infection (676.7 per 100,000) in Peru [23]. This is over five times higher than the national average, and is steadily increasing [23]. Loreto has one of the lowest densities of health workers in Peru, with a HRH density of 10.8/10,000 (compared to the national average of 17.8/10,000) [24]. There is limited research into the high mortality and HIV prevalence in Loreto. Past research has identified discrepancies between rural and urban health improvements in Peru due to poverty, low education levels, low sub-national governance capacities and underfinancing of the health sector in rural areas [25].

In 2015, the regional government of Loreto stated the need for “studies on the behaviour of patients with HIV and the importance of adherence to treatment” as a health priority (Gobierno Regional de Loreto: Dirección regional de salud de Loreto: Identificacion de prioridades regionales de investigacion para la salud 2015-2021, unpublished). This study therefore aims to understand the behaviours of HIV-positive men who have sex with men, including adherence and access to care.

A scoping literature search on Medline, PubMed and Web of Science revealed limited qualitative research into the barriers HIV-positive men who have sex with men face in obtaining optimal care of their condition in Peru. All relevant qualitative literature identified in Peru was from Lima. In 2006 Curioso et al. conducted a qualitative study which outlined barriers and facilitators affecting ART adherence in HIV-positive subjects [26]. Barriers included ART side-effects, simply forgetting, inconvenience and fear of disclosure and stigma [26]. Facilitators included a fixed routine, understanding the need for compliance, seeing positive results, treatment knowledge and having faith in treatment [26]. A 2016 cross-sectional study found a high-level of stigma was associated with HIV, which was negatively associated with ART adherence [27]. A 2019 study showed that patient-elected treatment supporters helped people living with HIV take responsibility for their treatment, facilitated transfer of knowledge and provided emotional support for patients in Lima [28]. A recent longitudinal study has identified the need for pre-diagnosis psychological assessment in Peruvian men who have sex with men to identify those with maladaptive coping pre-diagnosis who are less likely to link to care [29]. To our knowledge there has been no research into the HIV-related knowledge level of the men who have sex with men population in Peru.

Existing qualitative studies with HIV-positive men who have sex with men in Peru focus on perceived stigma [30, 31]. Clarke et al. concluded that HIV-positive men who have sex with men in Lima perceive that notifying a sexual partner of their HIV status was to reveal stigmatising information about their sexual practices, and portrayed the participant as promiscuous [30].

A worldwide search identified 16 relevant qualitative papers in HIV-positive men who have sex with men in regard to barriers to care; the majority undertaken in Africa and Asia. Stigma relating to HIV status and sexuality was a recurring theme as a barrier to disease control [32–37]. Two studies in China identified barriers to care in men who have sex with men as negative coping and mental health, misconceptions of ART benefits, non-disclosure of HIV status and lack of trust in the health services [32, 33]. Facilitators included social support, reduced HIV-related stigma and specialised services [32, 33]. Public education and other measures to reduce stigma were widely recommended to improve healthcare access and adherence to care [32–37].

## Aims and objectives

This study aims to improve understanding of perceptions and experiences of HIV-positive men who have sex with men living in Loreto, in order to identify barriers to accessing and adhering to the HIV care continuum. This should contribute to improved access and adherence to treatment regimens and a reduction in HIV transmission, to ultimately reduce the morbidity and mortality in this population.

Aims will be met through the exploration of the following objectives:

- To understand the extent of knowledge of HIV, including transmission, importance of ART and safer sexual practices.
- To establish the facilitators and barriers to local care pathway access and adherence.

## Methods

### Study design and population

A qualitative approach was used to gain insight into the experiences and perceptions of HIV-positive men who have sex with men and healthcare professionals (HCPs) in regard to barriers and facilitators for HIV-positive men who have sex with men adhering to the care continuum.

Semi-structured interviews with HIV-positive men who have sex with men (Method 1) and a focus group of HCPs working clinically with HIV-positive men who have sex with men (Method 2) were conducted in Spanish January–February 2019. Both were facilitated by an experienced interpreter (ES)^1^.

### Setting

Iquitos - the bustling urban capital of Loreto, Peru’s largest region. This vast area of Amazonian jungle is predominantly connected by waterways for which residents use slow-moving water transport. Its estimated population was 437,000 in 2016 [38]. The unique region is geographically isolated from mainland Peru, with limited contact to metropolitan capital Lima. The use of alternative Amazonian medicines is common.

### Method 1. Patient interviews

#### Population and sampling

Participants were recruited from the HIV Department of the Regional Hospital of Loreto (RHOL), a main provider of ART to men who have sex with men in Loreto, and Algo Bello Para Dios, a shelter for those with HIV in Iquitos. Participants were purposively sampled for a variety of responses and to explore candidate themes, representing a range of ages and socioeconomic backgrounds [39].

Biological males with a clinical HIV diagnosis were recruited. Participants had self-reported past sexual relations with a man, spoke Spanish or English and lived in Loreto. Participants that did not consent to the full interview, lacked capacity or suffered from a health condition that affected their ability to interview were excluded.

After expert consultation it was decided not to actively approach transgender women to participate, as this group were likely to have had different experiences to the men who have sex with men population despite similarities in HIV prevalence [14]. The study title was presented to potential participants, with participation at their discretion.

#### Recruitment

Men waiting in the clinic or shelter were approached by the principal researcher (PR) and ES. Potential participants were informed of study details in private. Eligible participants were given time to read an information sheet and consent form. Recruitment continued until data saturation was met.

#### Data collection

Individual in-depth semi-structured interviews were chosen to enable sensitive information to be shared [40]. A demographic questionnaire recorded age, sexual behaviours, occupation, condom use, adherence to ART, risk-taking behaviour, sexual identity and education level.

Interviews were conducted by PR face-to-face in a private room at the RHOL and the Algo Bello Para Dios shelter, exploring perceptions, experiences and understating of HIV and how this influenced treatment adherence. Interviews were recorded on an encrypted device. Field notes were taken for context [41].

A semi-structured topic guide (see supplementary Table 1, Additional File 1) was used based on research objectives. The structure was influenced by a previous guide from a study investigating adherence in HIV-positive men who have sex with men in Portugal [42]. A culturally appropriate case study was used to ease participants into a potentially sensitive interview, based on advice sought from an objective published author who had experience with this key population [43]. Participants were encouraged to share their individual experiences and views. The guide generated a variety of responses surrounding access and adherence to care pathways. A pilot interview confirmed that the guide was culturally appropriate and clear, so was included in the analysis.

Real-time interpretation between English and Spanish was provided by ES. Indirect translations were noted and discussed after the interview [44]. Translation quality was independently confirmed in the pilot interview by a second researcher who is a native Spanish-speaker. Information sheets, consent forms and the topic guide were translated to Spanish and checked for accuracy and comprehension.

### Method 2. Focus group with healthcare professionals

#### Population and sampling

HCPs were purposefully sampled to gain a variety of insights and generate an information-rich focus group discussion, with variation in age, sex and professional status. Criteria for inclusion were: adult, working with HIV-positive men who have sex with men in Loreto, able to communicate in Spanish and living in Loreto. Exclusion was as for Method 1.

#### Recruitment

Professionals were approached by PR and ES in the RHOL HIV centre. Focus group details and times were explained. Participants were given an information sheet and consent form in Spanish.

#### Data collection

A focus group of HCPs were invited to partake in a discussion between care providers in the main HIV treatment centre in Iquitos. This adds understanding and context to the main patient data collection. A semi-structured topic guide was adapted from the patient interview guide to align key themes and topics (see supplementary Table 2, Additional File 1). Recorded participant characteristics were age, occupation, gender, sex and time working with HIV-positive men who have sex with men patients. The focus group was audio-recorded and field notes were taken.

Interpretation and translation accuracy checks were conducted as for Method 1.

#### Data analysis

After completion, ES transcribed the interview and focus group recordings verbatim in Spanish, and then translated transcripts to English. Accuracy was checked by the PR and a second Spanish-speaking researcher. Discrepancies and non-literal translations were discussed. ES gave cultural context. Interview data were analysed in an iterative process throughout data collection by PR with input from ES. The salient points were discussed and recorded immediately after each individual interview in English to determine if data saturation had been met, and to determine future sampling decisions.

A thematic content analysis was used [40]. Braun and Clarke’s 6-phase approach was followed; to become familiarised with the data, undertake inductive coding and develop/refine themes [45].

A second independent researcher blind-coded a random sample of three anonymised interview transcripts for triangulation (83% concordance). Coding differences were discussed for moderation; the coding list was updated accordingly. A third researcher blind-coded the focus group (100% concordance). Discussion with ES ensured themes were culturally appropriate. Study validity was increased through the use of deviant case analysis and through reflection of PR on their impact on the process.

#### Ethical considerations

Informed written consent was taken from all participants prior to beginning the interview or focus group. Informed verbal consent was obtained from illiterate patients. Focus group participants were asked not to mention any patient identifiable data and to ensure that content was not discussed after the focus group. ES and the second Spanish speaking researcher signed a confidentiality agreement prior to beginning data collection. Interviews were conducted in a safe confidential space.

The study protocol was adhered to. Data was stored in accordance to the University of Birmingham’s data protection policy [46].

## Results

Results were split into interview results (Results 1) and focus group results (Results 2).

### Results 1. Interviews

Twenty-two participants were interviewed (to reach data saturation). Two were excluded from data analysis as they were not able to fully answer the questions and terminated the interview early. Mean interview length was 54.40 min (range 23.14–114.35 min).

### Participant characteristics

All 20 participants were native Spanish-speakers living in Loreto. Average age was 34 (range 19–55). Three participants could not read. Interestingly, four participants identified as ‘female’ gender; a point explored in the discussion. One participant identified as heterosexual despite prior same-sex sexual activity. Ethnicity was excluded; as a category this did not translate or have meaning for participants. Tables 1 and 2 summarise their characteristics.

**Table 1:** Interview participant characteristics

Gender	
Male	15 (75%)
Female	4 (20%)
Na	1 (5%)
Age (years)	
18–27	9 (45%)
28–37	4 (20%)
38–47	3 (15%)
48–57	4 (20%)
Work status	
Unemployed	4 (20%)
Student	2 (10%)
Part-time employment	3 (15%)
Full-time employment	5 (25%)
Freelance	2 (10%)
Other	2 (10%)
Prefer not to say	1 (5%)
Na	1 (5%)
Education level	
Secondary school, primary school or no past education	15 (75%)
Post-secondary education	2 (10%)
University or above	2 (10%)
Prefer not to say	1 (5%)
Sexual identity identified	
Bisexual	6 (30%)
Homosexual	9 (45%)
Heterosexual	1 (5%)
Prefer not to say	2 (10%)
Na	2 (10%)
Relationship status	
Single	14 (70%)
Has a partner	1 (5%)
Living with partner	1 (5%)
Na	4 (20%)
Past sexual experience	
≥ 1 man	11 (55%)
≥ 1 man ≥1 woman	4 (20%)
Prefer not to say	5 (25%)
Condom usage	
Always uses condoms	8 (40%)
Sometimes uses condoms	9 (45%)
Never uses condoms	2 (10%)
Prefer not to say	1 (5%)
Participation in unprotective receptive or insertive anal sex	
Unprotected receptive anal intercourse	3 (15%)
Unprotected insertive anal intercourse	3 (15%)
Prefer not to say	9 (45%)
NA	5 (25%)
Involvement in dangerous practices	
Not involved	13 (65%)
Injected drugs	2 (10%)
Exchanged sex for money	2 (10%)
Na	3 (15%)
Adherence to treatment	
Rarely forgets treatment	8 (40%)
Often forgets a dose	2 (10%)
Never forgets a dose	9 (45%)
Na	1 (5%)

Summary of interview participant characteristics (*n* = 20), Characteristics description proportion n (%)

**Table 2:** Frequencies of participant correspondence with sub-themes

Theme, sub-theme	Cases concordant with sub-theme content	Number of deviant cases	Cases where sub-theme content is not mentioned in interview
**Understanding of HIV**			
Incomplete patient knowledge	20	0	0
Public misconceptions	16	0	4
**Stigma sequela**			
Discrimination at work	5	3	12
Patient depression	14	0	6
Active avoidance of HIV patients	14	0	6
Shame as a barrier to diagnosis	12	2	6
**Education strategies**			
Need for more accessible information	13	4	3
Engaging young people	7	0	13
**Accepting treatment responsibilities**			
Personal acceptance	9	0	11
Adopting a new lifestyle	17	0	3
Support structures	18	0	2
**Practical barriers to treatment adherence**			
Traveling to clinic	6	0	14
Side-effects of ART	5	0	15

Interview participant correspondence with sub-themes, *n* = 20

### Findings

Five key overarching themes were identified from the interviews, listed below. Each theme comprised sub-themes. Participants are identified by ‘P’ and their assigned number after their quotation.

**Theme 1: Understanding of HIV**

**Theme 2: Stigma sequela**

**Theme 3: Education strategies**

**Theme 4: Importance of accepting treatment responsibilities**

**Theme 5: Practical barriers to treatment adherence**

Participant correspondence to sub-themes is summarised in Table 2.

#### (Theme 1) Understanding of HIV

This theme revealed the limited patient and public knowledge surrounding HIV, including common public misconceptions that result in active avoidance of HIV patients.

##### Incomplete patient knowledge of HIV

All participants understood the importance of condom use in preventing sexual HIV transmission. More advanced understanding of transmission was demonstrated by few participants.

[HIV can spread by] *“having unprotected sex, blood transfusion, from one open wound to another, and the use of syringes.” P12*

There were distinct gaps in participants’ knowledge. Some mentioned solely sexual transmission. Others mentioned modes that are incorrect, such as transmission “*using sharp objects and even through mosquitoes ( … )” P14*

All participants understood the importance of following treatment, information that seemed to have been provided by the healthcare team.

*“The doctor tells me that I can have a long life if I take my medication.” P18*

Knowledge of specific complications of HIV was limited, with the main ‘complication’ being that the patients can die. Five participants mentioned immune compromise and feeling ill, but overall understanding of complications was vague. This is an area that could be incorporated into future patient education strategies for early detection of common complications (Theme 3).

*“I think that when you do not take the medication, you can have complications like a strong flu that can kill you.” P12*

All of the participants agreed with the case used in the topic guide that features a patient stopping ART when they start feeling better. Most either described a similar past experience or knew of friends that had done the same, believing that the virus had been treated. This was often despite being educated by the HCPs. Those that had left the care continuum usually returned after suffering complications.

*“I had been taking my medication for a year and I felt very good so I thought I didn’t have the virus [so patient stopped taking ART] ( … ) six months ago I had serious complications.” P15*

Three patients had experience of substituting ART with a traditional medicinal diet or plant-based alternatives, or knew of someone that had.

*“A member of my family died from HIV one week ago, he didn’t stick to his treatment because someone else told him to take vegetables, he went to the jungle to drink oje, it is a plant that cleans the blood, then he came back in a very bad state of health and days later he died.” P16*

##### Public misconceptions

All participants outlined common misunderstandings about HIV that they had experienced in their families, communities, or had themselves prior to their diagnosis. Eight participants outlined the misconception that HIV was always an acute, terminal diagnosis. Upon diagnosis many were not aware that there was a treatment for the disease leading to low mood and fear.

*“I did not know there was a treatment for this disease and I felt very sad, now I know there is treatment and I feel good.” P9*

In some cases, this affected treatment adherence; participants believed that medication was futile if they were going to die anyway and hence rejected their treatment responsibilities (Theme 4).

*“A friend told me it does not matter if I take the medication, I’m going to die anyway, that was one of the reasons why I decided to stop the treatment.” P15*

Four participants highlighted that many believe those with HIV look ill and malnourished. Some participants reported having unprotected sex if a partner looked ‘healthy’.

[Friends] *“were quite surprised* [about my diagnosis] *because they thought that a person with HIV is a person who looks bad, skeletal and is bedridden, then when they found out they said “wow” because I did not look sick.” P5*

Most participants were frustrated at the lack of public knowledge surrounding HIV transmission.

“HIV is the first stage of AIDS, and people do not know that, they think that they are going to get infected just by touching.” P10

Some had experienced beliefs that HIV was limited to certain ‘groups’, e.g.

*“Many people believe that this disease is only acquired by promiscuous people, sex workers and homosexuals and that’s not true.” P5*

Improvements in public education and public campaigns (Theme 3) could address these misconceptions in the wider population.

#### (Theme 2) Stigma sequela

A culture of stigma and discrimination was reported. This was split into four sub-themes describing the effect stigma and discrimination had on participants: active avoidance of HIV patients, patient depression, shame as a barrier to diagnosis and treatment, and discrimination at work.

##### Active avoidance of HIV patients

A culture of avoiding those with HIV was described by 14 participants, leaving them feeling socially rejected. This had an impact on the participant’s social, family and professional lives. Some felt confined to their house. One case featured past discriminatory behaviour from a HCP.

*“I felt discriminated when I had an accident, the nurse didn’t want to clean my wound because he knew I had HIV.” P16*

*“My sister didn’t want to come close to me ( … ) and every time they* [my sister’s children] *approached me their mum used to tell them not to do it because I'm sick.” P5*

Many participants felt isolated by the lack of understanding in the general population as highlighted in Theme 1, including the misconception that HIV is spread through saliva and touching, depriving patients of kissing or sharing drinks. This, again, could be addressed by improved educational efforts (Theme 3). Some reported family forcing them out of their house and friends moving away.

*“One day a boy with HIV asked for a glass of water and my uncle gave him a glass of water and then he threw the glass in the trash and that is not correct because HIV is not spread by saliva.” P15*

*“I told my niece, then my uncles, and every time I went to ask for help, they would throw me out of their house and I used to leave crying, and my friends do not touch me or talk to me now.” P8*

Fear of discrimination led to participants opting to withhold their HIV status; one had not disclosed his status to anyone.

*“I lie to people when they ask me what I have because they will discriminate against me.” P8*

When asked to think about the experiences of HIV-positive homosexual, bi-sexual and heterosexual men most participants felt these groups shared the same experiences, seeming to interpret the question in a purely physical sense. One participant added that:

“Our society will always prefer heterosexuals and single out homosexuals ( … ) I had to pretend for many years that I am heterosexual so that my family accepts me.” P5

Eleven participants attributed stigma and discrimination to ignorance and a lack of education, including instances where views had changed once people had received relevant education. This highlights the positive and direct impact education can have on patient’s daily lives and support networks (Theme 3).

*“Some of them used to tell me not to get close to their children and they used to tell me ugly things, but when they, the doctors, explained more about HIV their attitudes changed.” P5*

Two participants did not report experiencing discrimination. Notably, they had accepted their disease and had family support. Three other participants believed discrimination had reduced.

*“Many people are aware now* [of HIV] *and they don’t look at you badly or point you out because of the disease that you have.” P13*

##### Patient depression

A diagnosis of HIV had severe psychological implications for 14 participants. Experience of low mood was often stated as “depression”. Seven participants mentioned feeling suicidal; one had acted on this:

*“When I was alone I used to think a lot about it* [HIV diagnosis] *and I tried to kill myself.” P3*

Education, the help of the clinical psychologist and family support were important factors in overcoming low mood*.* Participants stated the importance of education upon diagnosis, as most participants’ poor mental health improved after realising there was effective treatment.

*“The first few days I wanted to kill myself, but thanks to the psychologist, I'm fine now.” P8*

Participants described feeling better emotionally once they were taking the medication.

*“When I started taking the treatment I got up and I felt good and I had more desire to live.” P13*

##### Shame as a barrier to diagnosis and treatment

Shame was identified as a barrier to care by 12 participants. This affected all aspects of the HIV care continuum: diagnosis, care seeking, clinic attendance and adherence to treatment. Participants stated that fear of disclosure impacted on HIV transmission in the community.

*“People are afraid of rejection and that's why they don’t say anything and they have unprotected sex ( … ) my partner didn’t tell me he had the disease until I discovered it by myself.” P5*

Testing was typically done when participants had symptoms, or had suspicions about their partner’s fidelity. Only one participant went for routine tests. This was avoided by others because of fear and shame.

“*People are afraid or ashamed and they do it* [a HIV test] *when they feel poorly, or when they want to know if they have HIV.” P8*

Once diagnosed, shame was described as a barrier to care access and remaining in treatment. Participants did not want to be seen at the HIV clinic or taking daily medication.

“*I was afraid and embarrassed, I didn’t want people to see me in the HIV area when I started the treatment.” P15*

*“The majority of people feel ashamed and that’s why they die.” P16*

Shame informed why some chose alternative treatment options, a path made more attractive by the widespread belief in alternative Amazonian medicine described in Theme 1.

[I had a] *“friend who told me that he was ashamed to go to the clinic and that is why he was following a plant based treatment ( … ) it's been almost three years.” P5*

Two participants did not report feeling ashamed of their diagnosis. They had a high level of disease acceptance, family support and disregard for those that might discriminate.

[Discrimination does not influence my disease control] *“because I don’t give it too much importance ( … ) they laugh at me, but I don’t care.” P7*

##### Discrimination at work

Four participants shared stories of being discriminated against at work; three reported discriminatory testing requirements in low transmission risk occupations. A negative result was needed to apply for/remain employed in these cases. Some reported experiencing verbal discrimination. These experiences had negative psychological effects:

*“I am a supervisor and I called over an employee and she discriminated against me for having HIV in front of many people, it made me feel very bad.” P5*

*“When you apply to a company they ask you for a HIV test ( … )* [and if positive] *they do not accept you at work ( … ) I felt depressed.” P10*

Work had a detrimental effect on disease control. Participants feared revealing their HIV status at work so could not take the time off, and/or had to travel away for work, which impacted on clinic attendance and became a practical barrier to treatment adherence (Theme 5). Consequently, few participants had jobs.

**“***I had to travel for work and that's why I left* [treatment for six months]*.*” P20

Contrarily, HIV did not always interfere with professional life; one participant had been actively encouraged to seek treatment by his colleagues.

*“My bosses know that I have HIV and they send me to the hospital to receive my medication. I am a cook assistant.” P17*

#### (Theme 3) Education strategies

Most participants described the need for improved public understanding, particularly in areas of HIV transmission and treatment. Participants believed education could aid HIV prevention, disease control and reduce discrimination.

##### Need for more accessible education

Participants recommended improved public education strategies to reduce the HIV epidemic.

[Local men are at risk of HIV] *“because they do not have enough information and they are not aware that they should use a condom.” P19*

This was also considered important in improving access to care:

*Researcher: “What can be helpful for men who have sex with men in accessing care pathways?”*

*“To take care of themselves and attend the small group educational meetings about this topic.” P2*

Education provision when patients were diagnosed was mentioned as important by 10 participants, particularly for psychological health, early acceptance and disease control.

*“I got depressed but after receiving the treatment and the visits to the doctor I started to feel much better.” P2*

It was acknowledged by seven participants that public education improvements can reduce stigma and discrimination (Theme 2). Some stated that this was important in improving disease control for patients.

[To help HIV-positive men who have sex with men access freely provided care pathways there needs to be] *“total awareness, but not only to the men who have sex with men community, but also to the whole society because discrimination and rejection affects many.” P5*

Participants stated the need for more education through alternative routes in addition to school, including: small group educational meetings, local events and providing a specific place to acquire information. This is particularly important as some people do not attend school in Loreto.

[If I wasn’t diagnosed] *“I wouldn’t have known anything about HIV ( … ) I never went to school.” P18*

Participants described that those in communities and villages faraway from Iquitos are at higher risk of HIV due to reduced information access and provision, resulting in unsafe sexual practices. This group also faces difficulties in accessing treatment due to their remote location (as described in Theme 5).

*“People who are far away from the city are more at risk of HIV infection* [than those in the city] *because they don’t have information about the topic, some do not even know what HIV is.” P12*

Five participants however stated that education had reached a sufficient level in Loreto

*“There is enough education. In secondary schools there are courses about diseases ( … ) not only here but in the villages too, they carry posters and talks. I always participate in small group educational meetings.” P11*

##### Engaging young people

Seven participants believed it was particularly important to educate young people. School education was identified as important, providing opportunity to challenge stigmatising beliefs and prevent future spread of HIV. Most were unsatisfied with the HIV education that they had received, wishing it had gone deeper than solely mentioning safer sexual practices.

*“More information should be provided in schools, but not only in secondary school, but also in primary school, teaching children how can it be spread, prevention, and how to treat a person with HIV. I think that young people would learn to respect from an early age and there won’t be much discrimination.” P12*

Educating young people that are involved in ‘party culture’ was acknowledged as important in prevention.

Some participants stated that parents give their children too much ‘freedom’, which can lead to risky behaviours and HIV transmission. Improvements in parenting were identified as key to changing young people’s behaviour.

*“There is not enough education. I think education should start at home, if parents teach their children from an early age about HIV, children would grow up knowing how to take care of themselves.” P13*

Two participants thought that there was sufficient information for young people at school and through social media. They believed that young people do not seek out available information and consider the consequences of unprotected sex.

*“There is a lot of information on the internet, in social media. I think it is* [the spread of HIV] *because of parties and alcohol, young people don’t measure the consequences and they have unprotected sex with girls, and homosexuals.” P16*

#### (Theme 4) Importance of accepting treatment responsibilities

The importance of personal acceptance in improving and maintaining disease control was a trend identified throughout the interview data. Adopting a new lifestyle and support from family, friends and the hospital aided patient acceptance.

##### Personal acceptance

The importance of personal disease acceptance was discussed in nine interviews, regarded to be central in: maintaining disease control;

[I attend clinic] *“because it is my responsibility to come and stay in treatment.” P10*

the participant’s psychological wellbeing;

*“No* [I am not worried about my disease], *I got diagnosed 13 year ago and I have accepted the disease.” P16*

and – as earlier – overcoming shame as a barrier to treatment (Theme 2).

Participants stated that the consequences of non-acceptance resulted in a lack of care-seeking behaviour and poor treatment adherence.

*“I think that it’s one of the main problems, people do not accept their diagnosis and therefore make decisions that endanger their health.” P5*

##### Adopting a new lifestyle

A culture of alcohol-use, parties, unprotected sex and commercial sex in Loreto was described by all participants. This was considered responsible for the spread of HIV in men who have sex with men, with one participant describing this as the “lifestyle” of men who have sex with men.

*“In bars, men offer money to homosexuals in exchange for sex and they accept. They sell their bodies for* [US] *50 cents.” P17*

Once diagnosed, participants stated that people with HIV had to choose whether to adopt a new “responsible” lifestyle and adhere to treatment, or continue a “disorganised” lifestyle and lose disease control. This concept was expressed by 17 participants. A “responsible” lifestyle as advised by HCPs included: avoiding alcohol and drugs, going to sleep at a reasonable hour, taking medication on time and eating healthily.

[HCPs] *“forbid us to consume alcohol, drugs, smoke, even a lot of sleeplessness - it’s bad for us because we do not have a strong immune system.” P5*

*“I used to forget my pills and I abandoned the treatment for one year, because I liked to go to the parties and drink with my friends.” P18*

One participant believed that these lifestyle changes had had a positive influence and seemed thankful:

*“Now I have an orderly and healthy life style, I don’t sleep late or drink as I did before, I am very careful and hygienic with food ( … ) HIV intervened in a positive way and now I have a better life style.” P12*

This all-or-nothing health belief seemed to adversely affect adherence in some cases, with respondents considering leaving the treatment in order to resume their social life.

*“Sometimes I want to leave the treatment because of my friends, to go to parties.” P7*

##### Support structures

The importance of support systems in HIV control was highly regarded by 18. Family support was described as particularly important in achieving adherence.

*“If it were not for them* [family] *I wouldn’t take my medication and I would already be dead ( … ) I came to Iquitos to receive the treatment because my family supports me here.” P6*

Family support was important for psychological well-being and dealing with discrimination. Friendships were also described as a useful support system to a lesser extent. When not supported, patients were emotionally affected; one participant cried during the interview.

[I have experienced] *“bad comments of people, my neighbours and discrimination in general, but I do not care because my family love me” P6*

*[On diagnosis] “I told my dad, he lives with his partner and when she found out they told me to leave the house ( … ) they reacted bad.” [Participant starts crying] P20*

The psychological support, motivation and education from the medical team was viewed as important throughout the interviews. The psychologist was cited as particularly helpful in providing facilitators to care.

*“I feel bad, I want to cry because of the rejection, but I have the help of the psychologist.” P8*

Participants benefitted from taking their family to be educated at the clinic (Theme 3). This commonly dispelled misconceptions that family and friends had, and enabled them to support the patient (Theme 1).

*“I decided to bring her* [my sister] *to the hospital, so the doctor would inform her about HIV and then she changed, now I have a normal life, in my family, work and with friends.” P5*

Religious belief and support from a social worker were central for two participants remaining in care.

#### (Theme 5) Practical barriers to treatment adherence

Individual barriers to treatment adherence included travelling to clinic and initial side-effects of ART.

##### Travelling to clinic

Six participants outlined the difficulties of travelling long distances from Amazonian communities to reach Iquitos in order to receive monthly treatment, impacting on treatment adherence. Some found it difficult to afford travel costs.

*“Yes* [I am concerned about my health], *because I’m not going to have money to go to the clinic and have my appointments with the doctor.” P18*

Some participants mentioned difficulties taking time off from work to travel long distances to clinic, resulting in people leaving the treatment.

[Leaving ART treatment] “*is common in some people, because they live far away from the clinic, they don’t live in Iquitos and it is very difficult to leave our work to come to pick up the medicine.” P6*

One participant suggested decentralisation of treatment services to reduce travel difficulties.

[Some people’s treatment is delayed] *“because of the distance, that is why the treatment must be available in all hospitals.” P11*

##### Side-effects of ART

A quarter of participants described the severe initial side-effects of ART as a common factor behind patients leaving the treatment.

*“Many times, I wanted to leave the treatment because of the side-effects it caused to me, but I had to get used to it.” P12*

Understanding that initial side-effects would subside facilitated treatment continuation. This information had been received from the doctor in all cases.

*“The first two days the treatment hit me very hard ( … ) but the doctor told me that they are positive reactions for us, that’s how our body should react.” P5*

### Results 2. Focus group

#### Participants

All five HCPs that were approached agreed to participate in a focus group. Two could not participate as their clinical work that overran leaving a final focus group of three.

#### Participant characteristics

Participants were current employees at the RHOL HIV department. Due to identification risk in a small sample their specific roles are not published. Staff all had experience (ranging from 1 to 6 years) of working with men who have sex with men HIV-positive patients. The mean average age was 45. All were native Spanish-speakers (characteristics Table 3).

**Table 3:** Focus group participant characteristics

Participant	Age group	Gender	Healthcare role
1	30–39	Female	Member of the nursing team
2	40–49	Female	Allied health professional
3	50–59	Male	Clinician

Focus group participant characteristics, *n* = 3

#### Findings

Two themes were identified from salient features of the group discussion. Participants were coded ‘FG’ + an assigned number for quotation. The theme ‘ART treatment avoidance’ was separated into sub-themes. Focus group duration was 30 min 29 s.

##### (Theme 1) ART treatment avoidance

Participants had observed multiple barriers affecting patient access and adherence to treatment, including: stigma and discrimination, difficulties in accessing treatment, taking plant-based medicine and social barriers to care.

##### Stigma and discrimination

The clinician explained that discrimination is so common and wide-spread that it has become part of Loreto culture, perpetuated by a lack of information.

[Discrimination is] *“basically cultural, for example, when I see the patients, the family comes and they ask me doctor, can we talk to him? Or will he be able to eat with us? You see? it is the lack of information about the disease.” FG3*

Participants explained that discrimination in Loreto stems from poor education. They described that the public are unsure of HIV transmission, with beliefs that the virus can be transmitted through touching or talking, leading to patient isolation and emotional stress.

“*If your neighbour knows* [you have HIV]*, they do not want to talk to you anymore (...) I had a patient who came devastated because he could not even hug his children, because some people told his wife that he can infect them.” FG3*

They stated that patients become discouraged in controlling their disease after experiencing discrimination.

**“***Patients do not access the healthcare system, they know their diagnoses, but they are afraid, and ashamed.” FG2*

[Patients] *“do not accept to come and risk being seen by someone that knows them ( … ) there are people who become discouraged, they do not care if they die.” FG3*

It was stated that some patients were severely affected by discrimination, resulting in cases of depression and suicide.

**“***This kind of discrimination affects the patient psychologically, they think that the treatment is not worthy, they get depressed and they commit suicide.” FG2*

The participants seemed frustrated that there is information available online if patients or the public wished to find it, but they do not engage. This results in the continuation of widely held discriminatory beliefs and patient isolation.

*“There is a lot of information on the television and the internet, but people do not read, they do not educate themselves. That is why they have these wrong ideas and they are isolated.” FG3*

##### Access to treatment difficulties

The hospital staff stated that patients receive high quality care from their team at RHOL, however care at community health centres was of low quality with long waiting times.

[A major barrier to men who have sex with men accessing care is] “*the quality of services ( … ) there are some situations of abuse to the patient in health centres, for example: making them wait a long time, the type of treatment they receive.” FG3*

The distance needed to travel and transport costs to get to hospital was reported as a barrier to care.

*“Sometimes they do not have two PEN* [equivalent to 60 US cents] *for their transportation and that's why they do not come.” FG3*

This problem appeared worsened by the limited number of regional ART providers.

*“There are many health posts here, but few are authorised to provide the medication.” FG3*

##### Alternative medication

The focus group stated that alternative traditional plant-based medicines from the Amazon rainforest was often chosen in place of, or in conjunction with ART. This information was not always voluntarily communicated by patients and was seen by HCPs as part of Loreto culture.

*“There are some patients who give up on treatment for two or three months and always have illogical justifications ( … ) and many times they do not return ( … ) it's a cultural aspect, many people take natural medicine, but they don’t say it.” FG3*

*Researcher: “What kind of natural treatment have you seen people use?”*

*“Garlic sacha* [*Mansoa alliacea*, garlic of the jungle], *cat’s claw* [*Uncaria tomentosa, tropical vine bark*], *catahua* [*Hura crepitans*, possumwood]*, mururé* [Brosimum acutifolium, tamamuri bark] *( … ) there are many other plants ( … ) then they return in poor conditions.” FG1*

##### Social barriers

Poverty, lack of work, alcoholism and drug addiction were considered barriers to treatment adherence. It was mentioned that some employers required a negative HIV test for acceptance, despite this being illegal.

*“Some employers do not accept them if they are HIV positive. For example, two days ago a professional patient came, he was already removed from two jobs, even though he is a good professional, I told him* [he could take legal action]*.” FG3*

##### (Theme 2) Community education improvements

Staff described education as important in reducing stigmatising views and misconceptions. Educating families to gain their support was deemed particularly important for patients’ psychological wellbeing and treatment adherence.

Participants believed lack of education was behind risky behaviours common in Iquitos, particularly in the younger population.

[In Iquitos] *“there is a lot of debauchery, many parties, you see that very young men and women, are in states of alcoholism and drug addiction and that happens because of lack of education.” FG3*

The allied health professional highlighted that some rural communities cannot access HIV information, perpetuating misconception and discrimination.

*“There are many places, communities where people do not know what HIV is because there is no education, television, and that is why discrimination occurs and they decide to separate family members.” FG2*

Participants conveyed the need for improvement in educational programmes, which community agents are responsible for. Particularly, community health workers were highlighted as providing incorrect information and misleading communities, leading to confusion surrounding HIV.

[Community health workers] *“don’t go to the communities, they do not give informative talks like before.” FG2*

An annual plan to improve prevention of common infectious diseases was proposed, focussing on community care workers. These information providers should be proficient in teaching and providing correct information. Political action could make an education campaign more impactful.

“*The establishments or community care workers should have an annual plan of projects for the communities, they should provide information about diseases such as HIV, tuberculosis, etc. Community care workers are responsible for educating, they must be people who know about the subject and they must teach with pedagogical methodology.” FG3*

Parental education was deemed important to ensure families could support HIV-positive children/relatives and educate their children themselves.

*“The family support is very important, people who receive support from their families overcome it* [HIV-related depression] *easily.” FG3*

## Discussion

This is the first detailed qualitative study investigating barriers and facilitators to optimal care of HIV-positive men who have sex with men in Peru. Five central themes were identified by 20 patient participants: understanding of HIV; stigma sequela; education strategies; accepting treatment responsibilities; and practical barriers to treatment adherence. Two key themes were identified by HIV-specialist HCPs: ART treatment avoidance and community education improvements. It is noteworthy that staff and patient themes closely correlated. Study findings are similar to comparable studies in other low/middle-income countries.

Patients had a limited understanding of HIV and public misconceptions about HIV transmission and severity were described. The latter may link to Loreto having the highest mortality rate in Peru [23]. These misconceptions have not been previously identified in published reports in Peru. Similar findings have been identified in Africa [47, 48]. The assumption that it is possible to tell if someone has HIV from their physical appearance increases risk of transmission by failures to implement safer sexual practices.

Practical barriers to care were heavily featured in the data, particularly in the focus group. Participants identified travelling long distances to clinic 1–3 times a month as a barrier to care. Travel was stated to be expensive and time-consuming. Weiser et al. identified financial constraints and travel to be a principle barrier to care in Botswana [49]. Interestingly, this was not a barrier in urban Lima [26]. Investment in more local clinic provision of ART, mitigating the time and cost implications of travel to a centralised clinic, should be considered in line with World Health Organization guidance to increase ART adherence [50]. Clinic-based reimbursement schemes could be trialled, as financial support has also been shown to improve non-adherence [51].

Discriminatory misconceptions in the patient’s families and local communities result in the isolation and rejection of HIV patients. Most patients felt ashamed of their disease and feared the social and physical consequences. This led to delayed HIV testing and dissuaded participants from accessing care, closely corresponding to findings in Lima and Guatemala [26, 37]. Addressing treatment adherence-related misinformation and providing access to social support in a culturally sensitive manner has been found to improve adherence by Simoni et al. [52]. Adherence clubs and short messaging services (SMS) have previously been shown to improve adherence in some resource poor settings [50, 53]. In Lima, SMS with reminders and motivational messages have been shown to be feasible and were associated with improved adherence amongst HIV-positive young people [54]. A digitalised appointment system could assist in providing this service on a larger scale in Loreto [54]. Instruments to measure adherence should be developed [55].

Stigma and discrimination should be tackled by the involvement of grass-roots HIV advocacy organisations and regionally coordinated education programmes, working to enhance community empowerment in men who have sex with men [56]. Patients should have access to appropriate social support. Anti-discrimination and protection policies and practices should be regionally reviewed by policy-makers and governmental leaders with input from men who have sex with men stakeholders [56]. Programmes inside and outside of the healthcare service should promote such policies and codes of conduct to create a supportive environment [56]. Progress should be monitored.

The occurrence of employment-dependent HIV testing was highlighted in both groups, despite this being illegal [57]. Reducing discriminatory practices in the workplace is necessary to ensure patients can continue to treat and financially support themselves. Clinicians should ask about employment and encourage reporting to enable anti-discriminatory policies to be implemented. Wider population-based education is needed to make this a socially-acceptable option for patients.

Worryingly, 14/20 participants reported significant low mood related to their HIV diagnosis (Table 2) and 7/20 reported a history of suicidal ideation. Poor mental health linked to men who have sex with men’s HIV diagnosis has been identified in low and high income countries, with strong associations with social exclusion and experience of verbal abuse [35, 58, 59]. A 2016 study using bi-directional counsellor-participant exchanges found that depressive and anxiety symptoms were common post-diagnosis in men who have sex with men in Lima [60]. These SMS exchanges facilitated care engagement, retention in care and provided emotional support [60]. SMS exchanges could be trialled in Loreto, as previously mentioned. Further research should investigate the prevalence of low mood and suicide to help improve the steering of mental health services and retention in care.

Most participants did not think that men who have sex with men were treated differently from other HIV-positive groups, contrary to what was expected based on a comparable Lima-based study [30]. However this could be because in Loreto culture a positive HIV-status is directly linked with a homosexual identity, such that all individuals living with HIV are presumed to be homosexual.

The transgender population have been found to be more socially marginalised with worse care outcomes than men who have sex with men [14]. This group was not excluded at recruitment, but all subjects were recruited as males, due to concerns about differences in patient experiences between these populations. Four included participants identified themselves as female on the patient characteristics sheet (Table 1). Though it would be unwise to second guess anyone’s self-identification this finding could tentatively be explained by misunderstanding/misinterpretation of the question, female self-identification or undisclosed gender transition. Future studies are needed to compare and contrast experiences and perceptions of different HIV-positive groups to improve service provision and treatment adherence.

Participants were enthusiastic about the need for improved public and patient education in both data sets. Of particular importance was improved understanding regarding modes of disease transmission and the efficacy of ART treatment. This should reduce stigma. Young people, families and communities outside of Iquitos could be prioritised with improvements in quality and provision of community education through community health workers. Past research has found that school-based education programmes are cost-effective in preventing HIV in men who have sex with men in Peru [61], so this model could be drawn on. Previous national research has shown that if patients commence ART through community health workers, treatment coverage is increased and mortality decreases [51, 62, 63]. In 2012 the World Bank identified the need for strategic implementation of more trained specialist community health workers dedicated to HIV prevention and education from a baseline of four full-time employees for the whole region [64]. Recommendations were partly based on community health workers in Brazil, where there are competency requirements for training, workers are fully integrated into the local health system and the scope of practice is mandated by federal law [65]. Improvements of regional community education programmes are a priority to address in this region of Peru. Changes in community health should be coordinated and regularly monitored to ensure that improved health outcomes are maintained.

Acceptance of the disease was considered important in both accessing care and treatment adherence. Family support and living a ‘healthy’ lifestyle helped achieve disease acceptance, factors that were encouraged by the healthcare team at RHOL. The importance of disease acceptance and family support was unsurprising, being a common finding in the wider literature [26, 32, 36]. Some participants had experienced severe rejection, also found in previous research in India which had marked detrimental effects on adherence [66]. Public education campaigns should therefore ensure families are involved to improve patient support.

One barrier to adherence that did not align with existing international qualitative research was the culture of using traditional Amazonian plant-based medicine as an alternative to ART. The use of Amazonian plant medicine is well documented, but how this relates to HIV has not been specifically studied [67, 68]. Further research should investigate the prevalence and effects of alternative treatment use and HCPs should ask about alternative medicine use in routine clinic visits.

### Limitations

This study’s major limitation of recruiting from the RHOL and the HIV shelter meant that non-adherent patients or people that have not accessed care are not represented. We attempted to recruit this group but were informed by the host in Iquitos that it was not possible on account of there being no tracing system or recorded contacts for patients. This makes the study less generalisable but is a feature of such research in that only visible or traceable participants can be interviewed.

Meaning can be lost in translation. As far as possible interpretation risk was minimised by: discussion of unclear or non-literal translations, gaining cultural insight, translation accuracy checks of the pilot study and of transcriptions, further redacted translation with a supervisor and interpreter continuity in collecting and transcribing the data [44].

Validity of results in studies of this type can be increased by participant validation. This was not deemed feasible by the host due to illiteracy, the location and burden on participants.

Professional hierarchy may have influenced the discussion in the focus group.

Being a white, English, female student researcher gave the PR an ‘outsider’ position in the process [69]. Patients could have felt uncomfortable disclosing sensitive information to an outsider, potentially resulting in caution over sharing information. Conversely, they may have found it useful to talk to someone objective outside of their community and culture [69]. Participants seemed warm and open; no adverse effect was reported.

## Conclusion

Bringing about behaviour change for the purpose of HIV control is a complex, multifactorial process, involving: culture; issues of inequality and lack of empowerment; effective health care; the law and politics [70]**.** Against this background, this study shows that a lack of public knowledge of HIV is fuelling a culture of avoidance and fear of HIV-positive men who have sex with men. Discrimination poses a substantial barrier to HIV testing, access to care and treatment adherence. Culturally appropriate, evidence based, large scale educational campaigns are likely to help increase awareness of HIV mortality and dispel misconceptions that perpetuate discriminatory beliefs. Regional action is needed to provide high-quality education to communities. HIV should be presented as a treatable disease and transmission modes should be emphasised to change stigmatising beliefs. Parents and young people should be educated as a matter of priority. Work-based discriminatory policies should be identified and corrected in accordance with the law. Clinic-based travel reimbursement schemes should be trailed. The role of media and of non-governmental organisations, including charities, community groups, business and faith based bodies could be explored.

Societal acceptance of participants is likely to reduce experienced and perceived stigma of participants, increase treatment adherence and may help to reduce HIV mortality and morbidity in HIV-positive men who have sex with men in Loreto. Mental health management in HIV-positive men who have sex with men is a key priority.

## Supplementary information

**Additional file 1.** Interview and focus group topic guides.

## Data Availability

The datasets used and analysed during this study are available from the corresponding author on reasonable request.

## Abbreviations

HIV: Human immunodeficiency virus
ART: Antiretroviral therapy
UNAIDS: The Joint United Nations Programme on HIV and AIDS
AIDS: Acquired immunodeficiency syndrome
HRH density: Human resources for health density
HCPs: Healthcare professionals
RHOL: Regional Hospital of Loreto
PR: Principal researcher
P: Participant
US: United States
FG: Focus group
PEN: Peruvian nuevo sol (currency in Peru)
SMS: Short messaging services

## Footnotes

1 Interpreter held an English degree from the National University of the Peruvian Amazon.
