# Supplementary material for: HIV understanding, experiences and perceptions of HIV-positive men who have sex with men in Amazonian Peru: a qualitative study
Source: BMC Public Health. 2020 May 19;20:728. doi: 10.1186/s12889-020-08745-y (PMC7238527; doi:10.1186/s12889-020-08745-y)
Supplement: Supplementary file 1 — Additional file 1. Interview and focus group topic guides. [file 12889_2020_8745_MOESM1_ESM.docx]

**Interview topic guide (table 1)**

| Topic | Sub-topic | Probes |
| --- | --- | --- |
| Knowledge and experiences of HIV transmission, awareness of safer sexual practices and HIV care and treatment | Introductory case vignette | Raúl is diagnosed as seropositive in a HIV test. Initially he feels very sad and worried. He decides to receive antiretroviral treatment and with the treatment he feels better. He then decides to leave the treatment because he feels much better, and a friend of his recommends a special diet he could follow and not need the antiretroviral treatment anymore. A while later he becomes unwell and decides to see the healthcare professionals again. He had suffered serious complications of HIV that could have been avoided if he had not left the treatment.  Do you think that this case of HIV is common in Loreto? |
|  | Knowledge and experience of HIV | What do you understand about HIV as a disease?   - Do you know the ways that HIV can spread? - Do you know how HIV can be tested for? - Do you know the serious complications of HIV?   Does being HIV-positive interfere with your daily life?  Are you concerned about your health?  Do you ever feel upset or depressed as a result of your diagnosis?   - Did your mood improve? Why did it improve? |
|  | Knowledge of HIV prevention strategies | How can you prevent the spread of HIV?   - Do you use condoms when having sex? - If yes, how often? - If no, is this/are these sexual partner/s HIV-positive or not?   When is HIV usually tested for?  Why do you think that HIV continues to spread in Loreto? |
|  | Knowledge and experience of the local care pathways and treatments | What do you know about the local healthcare for HIV?   - What was your personal experience of being diagnosed? - What was your experience of being treated? - What was your experience of check-ups and regular care?   What do you know about treatment for HIV?  Have you been informed about the consequences of missing your HIV medication?  Do you think that there is enough education about HIV in Loreto?   - In what form is the education? - What education have you received? |
| Barriers to local care pathways | Perceived barriers | What do you think are the barriers for HIV-positive men who have sex with men accessing local care pathways?  What barriers have you experienced? |
|  | Perceived facilitators | What do you think can be helpful in HIV-positive men who have sex with men accessing local care pathways?  Why do you attend the clinic?  Have you had any experiences that have helped you access or stay in care? |
|  | Risk perception | Are local men in the area at risk of contracting HIV?   - Why are they at risk? |
| Reasons for delayed HIV treatment and poor adherence | Delayed treatment and poor adherence | Why do you think that some people’s HIV treatment is delayed?  Why do you think some people do not stick to their HIV treatment or clinic visits? |
|  | Patient experience of stigma | Who did you tell/not tell when you were first diagnosed?   - How did they react? - How did others react?   How do you think that the experience of being homosexual or bisexual and HIV-positive differ from the experiences of HIV-positive heterosexual men?  Have you experienced anything differently than you would have if you did not have HIV?  How does being HIV-positive interfere with your social, family, or professional behaviour?  Have you ever felt ashamed of your disease?   - Does stigma/discrimination have an effect on your disease control?   How do you think the people around you deal with you being HIV-positive?   - Do you think that there is discrimination against HIV in Loreto? - Do you think that stigma/discrimination has an effect on the access to treatment and disease control of other men who have sex with men in Loreto?   In your opinion, how can one fight stigma against HIV, specifically among men who have sex with men? |
|  |  | Thank you for answering these questions. Is there anything else that I haven’t asked about that you think would be important or interesting for me to consider? |

**Focus group topic guide (table 2)**

| Topic | Sub-topic | Probes |
| --- | --- | --- |
| Patient knowledge and education on HIV transmission, awareness of safer sexual practices and HIV care and treatment | Introductory case vignette | Raúl is diagnosed as seropositive in a HIV test. Initially he feels very sad and worried. He decides to receive antiretroviral treatment and with the treatment he feels better. He then decides to leave the treatment because he feels much better, and a friend of his recommends a special diet he could follow and not need the antiretroviral treatment anymore. A while later he becomes unwell and decides to see the healthcare professionals again. He had suffered serious complications of HIV that could have been avoided if he had not left the treatment.  Do you think that this case of HIV is common in Loreto? |
|  | HIV testing and treatment | When do people get tested for HIV?  Why do patients attend the clinic?  What do you think can be helpful in HIV-positive men who have sex with men accessing local care pathways? |
|  | Patient knowledge / education / and HIV prevention | Why do you think that HIV continues to spread in Loreto?  What is the level of education that people get about HIV in the community?  What education do they get once diagnosed?   - Is this enough?   Do people use preventative methods?   - Are condoms freely available? |
|  | Knowledge and experience of the local care pathways and treatments | What do you think are the barriers for HIV-positive men who have sex with men accessing local care pathways?   - Why do you think that some people’s HIV treatment is delayed? - Why do you think some people do not stick to their HIV treatment or clinic visits? |
|  | Risk perception | Are local men in the area at risk of contracting HIV?   - Why are they at risk? |
| Patient discrimination | Patient experience of stigma | How does being HIV-positive interfere with patient’s social, family and professional life?  Do you think that stigma/discrimination has an effect on the access to treatment and disease control of men who have sex with men in Loreto?  In your opinion, how can one fight stigma against HIV, specifically among men who have sex with men? |
|  |  | Thank you for answering these questions. Is there anything else that I haven’t asked about that you think would be important or interesting for me to consider? |
